# Evidence of Psychological Manipulation in the Process of Violent Radicalization: An Investigation of the 17-A Cell

**DOI:** 10.3389/fpsyt.2022.789051

**Published:** 2022-02-23

**Authors:** Irene González, Manuel Moyano, Roberto M. Lobato, Humberto M. Trujillo

**Affiliations:** ^1^Department of Psychology, University of Cordoba, Cordoba, Spain; ^2^Department of Psychology, Marbella International University Centre, Marbella, Spain; ^3^Department of Methodology for Behavioral Sciences, University of Granada, Granada, Spain

**Keywords:** psychological manipulation, terrorism, violent extremism, recruitment, violent radicalization, 17-A cell

## Abstract

**Introduction:**

Radicalization leading to violence is a complex social process that frequently targets young people. In this study, we examine the 17-A cell, which carried out terrorist attacks in the Spanish cities of Barcelona and Cambrils on August 17, 2017. We focus on the psychological manipulation techniques used to radicalized members of the cell.

**Methods:**

Using deductive content analysis, we examined the judicial order of the National High Court related to “Operation Ramblas” and the police proceedings of Cuerpo de Mossos d'Esquadra (CME) associated with the Barcelona and Cambrils attacks. Our goal was to determine whether psychological manipulation was used on the cell members and, if so, how frequently.

**Results:**

Our results suggest that different psychological manipulation techniques were used on the 17-A cell members to facilitate their use of ideological violence. The most frequent strategies were cognitive control (control of attention, group identification, and denigration of critical thinking), environmental control (control of information), and emotional control (authoritarian leadership).

**Conclusions:**

This study provides evidence that psychological manipulation techniques were used in the radicalization of 17-A cell members. The results are discussed in the context of previous research on the psychology of violent extremism and terrorism. We highlight the need for prevention and psychosocial interventions to steer young people away from violent extremism.

## Introduction

Violent radicalization poses a growing concern for European societies. In recent decades, cities like Barcelona, Berlin, Brussels, London, Nice, and Vienna have been targeted by political-religious terrorism. According to the 2020 European Union Terrorism Situation and Trend Report (1), in 2019, there were 119 attacks and more than 1,000 terrorism-related arrests in EU-member states. Among the perpetrators, approximately 70% were between 20 and 28 years old and roughly 60% were citizens of the country where the attack took place. Given these trends, it is necessary to study terrorism from a global perspective, with the aim of understanding the multiple motivations that lead young people to engage in political-religious violence.

In this research, we examine a topic that has been understudied in the terrorism literature: the use of psychological manipulation techniques in the process of violent radicalization (2). Research has found that young people are particularly vulnerable to propaganda and the influence of extremist recruiters (3, 4). Understanding how recruiters and groups use these techniques to radicalize youth is imperative to create effective prevention programs.

The present research focuses on the members of the cell that carried out the attacks on Barcelona and Cambrils on August 17, 2017 (commonly referred to as the 17-A attacks), which killed 16 people and injured several others (5–7). The cell that perpetrated these attacks consisted of nine young people and a leader (Abdelbaki Es Satty) who contributed to their violent radicalization (8). Therefore, the aim of this research is to shed light on the use of psychological strategies of manipulation in the violent radicalization of the 17-A cell members.

### Process of Radicalization

Radicalization is described as a “social and psychological process of incrementally experienced commitment to extremist political or religious ideology” (9). This process is unique to each individual although common recurring features can be identified. In recent years, terrorism researchers have moved away from a profile-based understanding of radicalization to focus on the different pathways leading to radicalization (10). This has led to greater attention on group factors and dynamics (11)—including social identity (12, 13) and social networks (2, 14–18).

Although group dynamics appear to play a crucial role in the radicalization process, there are important blind spots in our understanding of this complex phenomenon. One of them is the extent to which recruiters facilitate radicalization leading to violence. While some authors posit that recruiters play a minimal role—referred to as “bottom-up” radicalization (12)—others suggest a top-down process whereby the recruiter's role is more central due to their use of psychological manipulation techniques (19, 20). There seems to be evidence for both perspectives (21, 22). And yet, when research suggests a top-down approach, the manipulation strategies used by recruiters are typically unclear (2).

Recent theorizing on manipulation techniques, like the psychosocial model of recruitment and violent mobilization (19, 20, 23), suggests that the process of radicalization follows several phases, during which the recruiters indoctrinate and prepare young people for the use of violence. According to this model, recruiters identify their targets in vulnerable contexts—such as marginal neighborhoods, education centers, or places of worship. Recruiters then befriend their targets to build trust. As soon as the recruiter is accepted by the young person, he or she starts the radicalization process, which consists of three phases. The first phase is psychological submission (emotional radicalization), whereby the young person loses their autonomy and becomes dependent on their friendship with recruiter and cell members. This is achieved by using persuasive and aggressive communication strategies, such as social isolation and inducing confusion between reality and fantasy. The second phase is political-religious indoctrination (doctrinal radicalization), whereby the recruiter induces a new ideology using psychological manipulation techniques. Some of these techniques appear to be like those used by totalitarian cults and are aimed at eliminating the personal identity of the target by reinforcing a new social identity with the extremist group (24). Finally, in the third phase of violent disinhibition and legitimization (violent radicalization), the recruit validates the use of violence by associating with the mistreatment and oppression allegedly suffered by their new group, identifies the enemy, and shifts responsibility by making an attack essential to improving their situation (19, 20).

Providing evidence for this model, some studies have found a relationship between radicalization and the use of different psychological manipulation strategies. For instance, researchers have concluded that, in terrorist groups like al-Qaeda, there can be high levels of “group psychological abuse” similar to or higher than those of some cults (5, 24, 25), which use persuasion and manipulation to influence attitudes and behaviors, with the ultimate goal of inducing participation in terrorist acts.

### Youth and Violent Radicalization

According to EUROPOL's annual report (1), the majority of those arrested for terrorist offenses in the European Union during 2019 were young people between 20 and 28 years old. In Spain, 45% of radicals arrested for recruiting young Muslims to commit acts of political-religious terrorism targeted adolescents under the age of 18 (26). These data are consistent with previous analyses and in line with other systematic reviews that have emphasized that youth is a particularly relevant stage in the radicalization process, and as such, prevention strategies must focus on young people (20, 27–30).

Looking more closely at the radicalization risk factors presented by young people, Adam-Troian et al. (3) consider three specific components: (1) the experience of more extreme and variable emotions, (2) greater threat/stress sensitivity, and (3) commitment with violence. According to this perspective, adolescents experience more extreme and variable emotions given deficits in both emotional regulation and emotion reactivity (sensitivity) (4, 31). Likewise, young people are more sensitive to grievances and peer influence, especially social rejection (32). Indeed, peer ostracism outweighs other negative decision outcomes (33). In the same way, young people can be particularly sensitive to propaganda efforts from violent extremist organizations' recruiters (34), engage more frequently in risky behaviors (35) and commit more violence than other age groups (36). Males are more prone to aggressive behavior that females due to both biological and socialization factors (37).

In conclusion, because some developmental characteristics make youth more sensitive to threats that can lead them to engage in political violence, age could be considered a risk factor for radicalization (38). In this context, the religious knowledge of recruiters may be a factor of interest and admiration among young Muslim Europeans of the second and third generation, who typically lack religious training, adaptive social models, and critical thinking skills. Providing evidence, Schmid (39) found that some youths who join political-religious terrorist groups “know little of Islam, that their knowledge is “a la carte”—an eclectic mix of out of context Koran and Hadith quotes gained from websites rather than acquired from a study of more trustworthy sources” (p35).

Given these risk factors, it is clear that the aim of recruiters is to lead young people to emotional and cognitive states that facilitate violent disinhibition (24). Therefore, to better understand the process of youth radicalization, greater attention must be paid to the interaction between young people, potential recruiters, and group dynamics.

### Psychological Manipulation

Psychological manipulation is a directed and systematic process carried out by an indoctrinating hierarchy with the application of specific techniques and strategies (5, 40–42). Manipulation is contemplated as something subtle, imperceptible, and gradual without the application of violence or force. Defined in this way, individuals are unaware of the manipulation, which undermines their volitional capacity (43).

Some scientific studies have compared terrorist dynamics with those applied in cults, highlighting certain similarities between the two. Trujillo et al. (24) evaluated the psychological manipulation processes that take place within terrorist groups, showing the use of these dynamics by certain leaders. According to Cuevas (44), terrorist and cultist dynamics are nearly identical, with the only difference being the use of fear and violence by terrorist groups as a means of self-assertion. In any case, the possibility that terrorist groups use this type of technique has not been studied in detail.

By collecting the previously identified psychological manipulation techniques from cult victims (42, 45, 46), Cuevas, using the Coercive Persuasion Interview (CPI), proposes a new classification to group them (40). He highlights four groups of manipulation strategies. The first is formed by environmental techniques with the objective to distance the subject from their previous environment and routines, promoting immersion in the group and restricting their freedom or will. These techniques include isolation, control of information, creation of a state of existential dependence, and psychophysical weakening. The second group includes emotional techniques aimed at influencing the feelings and emotions of the subjects to generate greater submission to the group. Among these techniques we find emotional activation of joy, activation of fear, guilt, and anxiety, as well as rewards and punishments. The third group comprises cognitive techniques used to confuse and alter mental processes. Among these techniques are denigration of critical thinking, lies and deception, demand for condescension and group identification, control of attention, control over language, and alteration of sources of authority. The fourth group includes techniques to induce dissociative states, which have the aim of altering general awareness or behavioral, cognitive, and emotional inhibition/disinhibition. Methods in this group include use of drugs, refusal of treatment, medical help and/or refusal to take prescribed medication, chanting, mantras, speaking in tongues, praying, meditating, and other methods of dissociation such as suggestion and hypnosis.

In combination, these techniques are used to distance individuals from relatives and friends, generate peer pressure, and override individuality and critical judgment (40, 42). In other words, the autonomous will of the individual is conditioned or overridden in order to accept and satisfy the will of the group (40, 47).

### The Present Study

Based on the information presented above, the aim of the present study is to provide evidence for the top-down radicalization process and manipulation techniques used within a terrorist cell. This study focuses on two research questions: (1) Were psychological manipulation techniques used to radicalize young people into the 17-A cell? And, if so, (2), Which techniques were used most frequently? To answer these questions, we conducted a systematic analysis of the radicalization process of the members of the 17-A cell.

## Methods

To answer the research questions posed, we designed a case study based on the 17-A cell. The 17-A cell was formed in the municipality of Ripoll (Gerona, inner Catalonia) and included 10 members: the leader, who drove recruitment (imam), and nine young people among them there were four pairs of siblings among the members of the cell, each of whom were second generation migrants (48). The age of the members ranged from 17–28 at the time of the Barcelona and Cambrils attacks; four were not over 20 and one was under the age of 18. All of them were residents of Ripoll, and most shared family or friendship bonds within the group. According to different analyses, the leader, allegedly inspired by the jihadist Salafist doctrine Takfir wal-Hijra (5, 49, 50), was a key element in facilitating the radicalization process by bringing the other members together.

While the cell intended to carry out larger attacks using three vans loaded with explosives at neuralgic points with a high influx of people (8), it had to improvise after their operating base in the municipality of Alcanar (Tarragona) exploded, killing two members, including the recruiter and leader (8). Following this operational mistake, the group, without a clear figurehead, took the following actions: On August 17, 2017, faced with the impossibility of carrying out the original plan, decided to establish the city of Barcelona as its initial target. Once there, one of the members drove a vehicle into the well-known pedestrian street of Las Ramblas (one of the most crowded streets in the city) killing 14 and injuring a large number of people (8). During the escape, this same person killed a young man with a knife. The next day, in Cambrils, a town frequented by tourists in the summer, five members of the group drove a car into a seafront promenade, running over pedestrians. They then exited their vehicle and attacked pedestrians with bladed weapons acquired hours earlier in a bazaar. One person was killed and several others injured before the terrorists were shot by police (8).

According to the testimony of citizens in Ripoll, the perpetrators were “apparently integrated” young people (6), meaning they were perceived to have access to good schools and jobs, participated in their communities, spoke the local language, and except one of them, had no previous criminal records. The alleged recruiter, had established contact with these young individuals in 2015, 1 year before the attacks, when he served as an imam in a local mosque in Ripoll. According to the legal resources consulted (51, 52), although the mosque was the place where he initially contacted the youth, the subsequent meetings were held in the recruits' homes. It was during these meetings where Es Satty acted as the primary radicalizing agent and guided them toward an exclusionary and belligerent view of Islam that facilitated the legitimization of violence.

### Materials

To test our research questions, we compiled public official documents relative to the 17-A attacks in Barcelona and Cambrils. The first document used was the Summary Judicial Prosecution Order No. 5/2018 (51), dated October 10, 2018, of the Central Court of Instruction Number 4 of the Spanish National Court. This 43-page document was written by Most Illustrious Fernando Andreu Merelles, Magistrate-Central Examining Judge Number 4 of the National Court, based in Madrid, Spain. The document is open source and subject to the judge's interpretation of the evidence gathered during the investigation. The second document is the police proceedings number 680566/17 (52) relative to “Operation Ramblas” led by the Mossos d'Esquadra (CME) police force on December 10, 2018. This document consists of 101 pages and was co-written by the police instructor with Professional Identification Card number 8261, and the police secretary with Professional Identification Card number 7603. Both documents are open source and subject to interpretation, but the systematic procedures of the system help improve the impartiality of these data sources.

### Analytical Procedure

To analyze the compiled documents and search for evidence of psychological manipulation, we used a deductive method of content analysis (53), which made it possible to identify the frequency of appearance of different strategies and techniques. To this end, an information gathering instrument was designed, based on the CPI model developed by Cuevas (40).

The CPI is a semi-structured interview instrument for the evaluation of psychological manipulation of groups and cults. The interview script incorporates 66 questions that seek to identify the presence of 17 manipulation techniques categorized in four groups of manipulation strategies: environmental, emotional, cognitive, and dissociative. In Spain, the CPI has been applied in forensic processes related to victimization of abusive groups (40). To adapt it for our analysis of the 17-A cell, all questions included in the CPI were reviewed by two experts from the research team, eliminating those that were cult-specific and adjusting others to the terrorism context. Using this approach, we were left with 14 manipulation techniques clustered in four groups: strategies of environmental control, emotional control, cognitive control, and inducing dissociative states (see Table 1). For the coding of the data, a record sheet was designed to list the indicators found, the page number where it was detected, and the total number of locations of that indicator.

**Table 1:** Strategies, techniques, definitions, and indicators of psychological manipulation adapted from the CPI.

**Strategies**	**Techniques**	**Definitions**	**Indicators**
Environmental control	Isolation	Separating or distancing the group member from his/her environment of relationships, spaces, routines or usual habits, promoting his/her immersion in the vital space of the group.	Termination of connections with family members and/or friends to join the new group of friends. Change of routines of the usual activities of the person (such as studies, work, and sports).
	Control of information	Selection and management of information, including the devices for communication and training in the group, in favor of the interests of those who control the group and aim to monopolize the information that reaches the individual.	Control of information checked by the members of the group. Limitation and control of the devices used for communication and training in the group.
Emotional control	Authoritarian leadership	Making the individual obey and grant maximum power and recognition of special qualities to a single source of authority that governs or inspires the governing of the group.	Behaviors of submission and obedience within the group that favor the ingroup cohesion. Hierarchical group structure: the leader sets the goals to be attained and the means to attain them. There is a leader who controls the interactions among group members and with himself/herself. The leader is viewed as a superior or divine authority who is full of knowledge and wisdom.
	Emotional activation	Recruitment maneuvers that help the individual to get attached to the group. Psychological manipulation techniques are used to control and dominate the person.	Use of psychological manipulation techniques for the recruitment process (thought reform, indoctrination, love bombing, environmental control, cheated). The group covers the affective or sexual needs of the members.
	Activation of fear of guilt	The individual is subjected to humiliation or guilt for past mistakes that have no connection with the group. The altered emotions of the individual are exploited to present the group as safe and beneficial, while showing the world outside the group as unsafe and that re-joining it will lead to failure or even death.	Use of guilt for past and/or present flaws and mistakes.
	Application of rewards and punishments	Attitudes or behaviors that are not consistent with the group are punished. The individual is criticized or corrected when he/she develops an unwanted behavior. Those who are loyal to the leader are rewarded.	Lecturing, punishment and exclusion if the ideology and/or rules of the group are not followed. Use of rewards within the group (martyrdom/paradise/veneration).
Cognitive control	Denigration of critical thinking	The members of the group must assume and accept the arguments through an authoritarian model in which the rules are followed without debate. Critical thinking is penalized; in this case, the reasoning of the leader is faithfully followed, that is, the hierarchical figure is absolutely trusted.	Reduction of cognitive dissonance through clear and final answers to the ambiguity and doubts of the members (cognitive closure). Prolonged exposure to ideologies or doctrines that hinder critical thinking, reflection, democratic values and/or respect for individual freedoms.
	Group identification	The group establishes an elitist or supremacist belief on the knowledge that is alien to any person outside of the group, in a way that the individual believes to be in a superior state that marks the difference between the outside world and the group.	The group is shown as cohesive and united, adopting a group identity. Conformation of a single way of thinking (group thinking). Use of symbols or aesthetics that promote identity among the members of the group. Closed group where the members become a second family. The group maintains a belief that is above everyone else.
	Control of attention	Programed activities are carried out to keep the members busy within the group, with no option to choose activities freely.	High participation and involvement in the activities, which consolidate the group support and commitment.
	Control over language	The way of expressing oneself is modified in a way that there is a change in how the members of the group think, through an altered vocabulary, new expressions, and neologisms.	Use of a language or jargon that is unique to the group.
	Alteration of the source of authority	There is a change in the authority figures of the individual to focus on a single authority, a single code of conduct, a single law dictated by the leader of the group and which will govern the behavior and rules of the individual.	The group doctrine is above everything else, and it is dogmatic and unquestionable.
	Perceived conflict	The doctrine and rules of the group are perceived by the individuals as the only possible choice. A polarized thinking is established, in which everything outside of the group is blamed for the suffering and injustice of the group members.	A dichotomous view of life is promoted, dividing it into two realities: “us” (group) and “them” (society, institutions…). The group prevents its members from identifying themselves with the general rules, beliefs, and values of society. Identification of the outgroup (society) as guilty or responsible for the grievance against the ingroup.
Inducing dissociative states	Use of drugs	Use of drugs that alter the use of general awareness or behavior, cognitive and emotional inhibition/disinhibition.	The use of drugs is allowed within the group for the attainment of its goals.
	Chants, meditation, praying	Cognitive control of the group members through chants, prayers and linguistic-emotional expressions that can imply a state of dissociation to lead the individual to a sense of unreality.	Use of chants that triggers strong emotions intended to persuade the individual about the ideology.

Using this tool, a trained coder evaluated the material. The minimum coding unit was one sentence. If the same topic was expressed in more than one sentence, the entire coherent segment was marked with the corresponding indicator. Similarly, when the code indicator was coded in different segments of the text of a single page, the segments were considered as different codes of the same indicator. Moreover, the indicators were not exclusive, thus, a single coding unit could have two or more indicators assigned. Subsequently, another coder reviewed all the extracts to determine whether they corresponded to the assigned indicators. When this second coder disagreed with the assigned indicator, the discrepancies were discussed with the first coder until agreement was reached. Finally, we calculated the frequencies and percentages of appearance of each of the variables according to the sum of the coded indicators.

## Results

**Figure 1:**
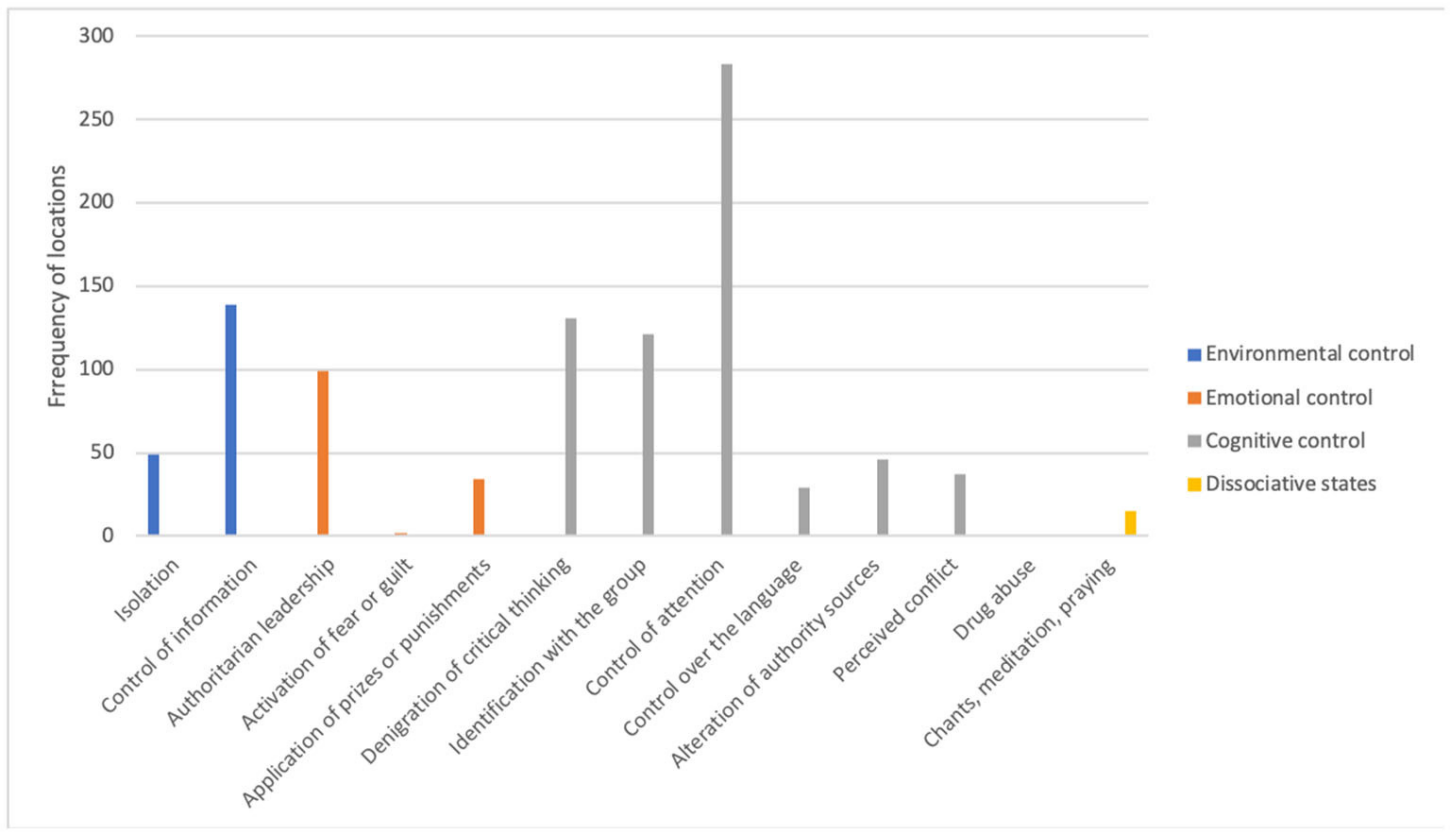
Evidence of psychological manipulation (strategies and techniques) in the 17A cell.

The results summarized in Figure 1 refer to the frequency of appearance for each technique of psychological manipulation found in the Summary Judicial Prosecution Order No. 5/2018 (51) and police proceedings number 680566/17 of the CME (52). Next, we present the combined results derived from both documents.

These results suggest that different psychological manipulation strategies and techniques were applied, facilitating the recruitment, indoctrination, and legitimation of violence by the young people who were part of the 17-A cell. The most frequently used manipulation strategies were those included in the cognitive control strategies (66%). Among them, the techniques control of attention (28%), group identification (12%), and denigration of critical thinking (13%) were the most prominent. The second group of strategies that appeared most frequently was that of environmental control (19%), with the technique control of information (14%) standing out. The third group in order of frequency was emotional control strategies (14%), with the authoritarian leadership technique being the most prominent (10%). Finally, the fourth strategy, corresponding to the induction of dissociative states (such as use of drugs, chanting, and meditation) had a low frequency in the data sources (1%) and is not elaborated here.

Cognitive control strategies were present through attention control techniques, techniques of group identification, and techniques of denigration of critical thinking. Regarding attention control, the leader used planned group activities to encourage commitment to and attainment of the group's goals, which gave the recruits a sense of purpose—a feeling of achievement, competence, and meaning (heroes, martyrs). These activities were logistical, spiritual, and related to the consumption of propaganda (51, 52).

The logistical activities encouraged the involvement of young people, which in turn encouraged pro-group behavior (54, 55). Some examples were finding information on the internet on how to manufacture explosives, and indoctrination meetings. For examples, the documents state: “In May and June 2017, Abdelbaki ES SATTY began to prepare the rest of the members of the group to commit the terrorist act by using the explosives they were preparing, telling them they would be martyrs” (A5, p17). Reports from indoctrination meetings are also explicit:

During these last months (before the attacks) the group would meet more assiduously, approximately two or three times a week in Ripoll (Girona) mainly in the street or in the mosque. The subjects that they approached in these meetings would be centered in speaking about the objectives of the attacks and about the use of explosives, atrocities and knives, justifying such acts since, according to what the imam Abdelbaki ES SATTY would have told them, to make this type of act was to be part of the Islam (A9, p17).

As for spiritual activities, these were realized through increased prayer and mosque attendance. The leader brought them closer to the faith (Islam) and their Muslim identity and therefore, to remember what they should and should not do (like avoiding Western behaviors, such as drinking or going to discotheques). For example, as one document noted: “Younes ABOUYAAQOUB, Youssef AALLA and Mohamed HICHAMY three years ago already started to modify their behavior as they stopped going out in groups and meeting to play soccer and stopped maintaining the close relationships they had maintained years before. The declarant himself related this to the frequency of attendance at the mosque” (D10, p11).

Regarding the consumption of propaganda, activities were carried out that included a massive consumption of jihadist materials that legitimized violence and self-sacrifice. As one document put it: “The content found in several of the electronic terminals of those investigated also reflects a profuse consumption of jihadist videos, predominantly DAESH productions, as well as the continuous listening of *anasheeds* that extol and encourage to achieve the goodness of the jihadist imaginary through martyrdom” (D1, p6).

For the group identification techniques, the coded data indicated that the leader managed to merge members' “personal self” with the “group self,” which promoted a feeling of belongingness that compensated for the conflict of identity and restored the meaning of individuality. This included: “The life of each one of them developed exclusively together with that of their cellmates, sharing housing, meals, trips and, therefore, participating in the necessary acts to carry out an attack against those considered “their enemies,” that is, the Western world” (D1, p10). The establishment of this exclusiveness model among the members fostered, on the one hand, relative distancing (from relatives and friends) and the progressive abandonment of previous routines, and, on the other hand, strengthened the ideas and beliefs of the group (shared values and norms), which increased their willingness to sacrifice themselves and participate in violence in the name of the group. With the latter dynamics, the leader activated the disposition of the members to fight for those shared values and beliefs that they considered sacred and non-negotiable, as denoted in the following observation:

The members of the cell present themselves as chosen by Allah, chosen among thousands of Muslims […] to defend Islam, using violence. In this period of time, they dispose of the necessary means to commit the attacks and they finalize the identification of the targets to perpetrate them looking for the maximum injurious capacity, understanding the jihad as an obligation and the murder as a norm (D8, p74).

Using denigration of critical thinking techniques, the leader motivated members toward extremist ideas using a bias view of the Holy Scriptures. For example: “Mohamed HICHAMY, Younes ABOUYAAQOUB and Youssef AALLA went to the imam's house after prayers and justified the use of violence in the name of Islam” (D23, p3). The frequent and prolonged exposure to violent radical material (such as Islamic State jihadist propaganda) was also used: “The massive and shared consumption of jihadist propaganda, mostly published by the production companies of the terrorist organization Islamic State-DAESH, has led to the rapid indoctrination of the youngest” (D1, p96). This established a unique and exclusive group thinking (56) based on the political-religious ideology that strengthened and validated the internal postulates of the group. The recruiter also used audio techniques, such as *anasheed* (chants) and audiovisual material from *sheikhs* within the Islamic State terrorist group, which could have contributed to the annulment of members' critical thinking, furthering an embrace of group dogma at the expense of autonomic thinking: The constant and repetitive listening of anasheeds inciting to martyrdom got the members of the local cell into a state of mind favorable to the sacrifice of their lives (D96, p1).

The second-most prevalent manipulation strategies, environmental controls, were detected through information control techniques (51, 52). Although the leader established some restrictions on the instruments used for group training and communication (computers, tablets, mobile), such control was not absolute since all the members had smartphones and other electronic devices with access to the internet. In this way, although the leader did not limit the access to information sources, he did guide and manipulate them toward the alternatives he wanted them to access, which may have played a role in reducing their critical thinking and inducing a greater need for cognitive closure. For example: “Youssef AALLA, Younes ABOUYAAQOUB and Mohamed HICHAMY would do everything that the imam Abdelbaki ES SATTY told them, who would provide them with the propaganda material of the terrorist organization DAESH on a tablet that they would pass to each other, which contained jihadist videos, among other contents” (A10, p4). The fact that the leader offered clear and simple answers to members' questions of doubt (57) could have favored the restoration of personal meaning as observed in this example: “In this last phase, the members of the cell are fully determined to perpetrate them [the attacks], under the auspices of the legitimacy granted by the figure of their emir or spiritual leader, the one exercised by Abdelbaki ES SATTY, impregnating the concept of martyrdom by Islam” (D4, p74). The leader's actions also prevented the appearance of discrepancies on the decisions taken by the group regarding logistical planning, as is observed in the following quote:

Driss OUKABIR's hesitation is argued for not making the logistical arrangements and not for making them against his will. Moussa OUKABIR answers him that this is “Shaitan (devil)” and tries to give courage to his brother for justifying the violent [extremist] actions of believers in Islam against apostates in the name of God, transmitting courage and condemning the one who runs away from this duty. “Read Anfal. Listen to me. That is the truth” (D10, p7).

Analysis of the documents suggested that the members of the cell were indoctrinated and radicalized in ideological precepts compatible with those of the group Jamaatu At Takfir wal-Hijra (5, 49, 50). The doctrine of this group is usually associated with three principles: (1) the obligation to fight against all those who do not submit to Islam, (2) migrating or isolating from the ignorant or ungodly in society, and (3) the Taqiyya (58). Taqiyya corresponds to the act of concealing or hiding one's beliefs when under threat, persecution, or coercion, or when these behaviors and activities serve the pursuit of group ideology and goals (e.g., jihad). Therefore, the application of this strategy by terrorist groups could allow them to carry out criminal acts that would be prohibited in Islam in order to finance their fight against the infidels, in which the end justifies the means (49, 59, 60). Based on the analyzed data, the Taqiyya doctrine may have been applied in the 17-A cell, allowing its members to fulfill their obligations as non-Muslims (5, 50). For example, in the cell, the youth carried out activities forbidden in Islam, such as squatting in houses (*gasb* in Arabic) to remain undetectable (*sura Al-Baqarah*): “The group decided to occupy a house in Gombrèn (Girona) where they would hold their meetings” (A20, p5). Somehow, this made it easier for them to adopt behaviors that would be forbidden without restraining themselves from certain previous habits, such as drinking alcohol, smoking, dressing in Western fashion, not having a beard, having sexual intercourse with Western women, using drugs, and skipping the mandatory prayers (5, 49, 50).

The third-most common manipulation strategies, emotional control, were detected through authoritarian leadership techniques. The leader set the goals and the means to attain them. With a spiritual role, the leader managed to guide group members in a biased view of their shared belief (religion). The vast knowledge of the leader in religious matters, and the youths' need for self-determination, made it easy to present his word as dogma and his presence as divine.

It is important to highlight that the leader did not create a narrative; that is, he did not generate ideas of his own. Rather, based his discourse on a distorted view of the Holy Scriptures, on which he applied a system of reflective logic to justify and exploit the vulnerabilities of the cell members. Thus, the leader controlled the interactions between and with group members, as evidenced by this observation: “Abdelbaki ES SATTY, adopting the security measures in telephone communications described in the chronology of the day 09/08/17, tried to communicate from his conspiratorial telephone […] with the personal telephone and the conspiratorial telephone of Younes ABOUYAAQOUB as well as with the personal telephone of Youssef AALLA” (D18, p50). He was also seen as a higher or divine authority with unique knowledge and wisdom: “Mohamed HOULI CHEMLAL ratifies the group relationship between the investigated and Abdelbaki ES SATTY, giving him the role of leader or emir” (D1, p23). And he demonstrated behaviors of submission and obedience within the group that favored ingroup cohesion: “The leader also established the goals to be achieved and the means to achieve them: the cell had its “Emir,” or leader, a figure given to Abdelbaki ESSATTY, who indoctrinated them in violence and convinced them to carry out a terrorist action” (D26, p95).

Nonetheless, despite the leader's intellectual role, the group adopted a network structure through collaborative participation without restrictions. Among the cell members, an information flow was established, which acted as a distributed network where the hierarchy was flattened and there was free connection between the members and the world outside the group. This can be seen in the witness statement made by someone close to two of the members of the cell, in which the declarant states that “the last time he saw Moussa OUKABIR and Said AALLA were on the sport facilities 4 days before the attacks” (D5, p14). Given that there were four pairs of siblings among the members of the cell, the leader also used these family and friendship relational structures to guarantee control, submission, and secrecy. This could be facilitated because the members shared similar circumstances, the same origin, and common values (religion), as can be observed in the following quote: “All of them had known each other since they were children, having grown up and become friends as they were all neighbors of Ripoll” (A26, p3).

Finally, there are two indicators included in the CPI instrument that were not found in the documents analyzed in this study: the use of drugs and the affective or sexual needs of group members. However, evidence of these indicators was found in other documentary sources (such as media reports) collected during the research process (61, 62). Thus, for the strategy to induce dissociative states, we found that the leader allowed the use of drugs as a facilitating element for behavioral, cognitive, and emotional inhibition/disinhibition toward the objectives of the group. This could enable certain members to maintain previous habits, contributing to their loyalty and group commitment. Likewise, for the emotional control strategy, the leader used recruitment maneuvers such as covering the affective or sexual needs of the youths by allowing them to go to brothels or to keep certain previous romantic relationships. Moreover, this may have minimized defection from the group by allowing activities that would help satisfy members' emotional needs.

## Discussion and Conclusions

Although the group dynamics involved in radicalization constitute a broad field of study, aspects such as the use of psychological techniques of manipulation have received less attention. The aim of this study was to provide evidence for the top-down radicalization process that occurred in the 17-A cell and to document the type of psychological manipulation techniques that were used to radicalize its members.

Our results indicate that several manipulation techniques were used on cell members by its leader. The most frequent strategies were cognitive control (control of attention, group identification, denigration of critical thinking), environmental control (control of information), and emotional control (authoritarian leadership). The results reveal the hierarchical use of manipulative strategies to facilitate recruitment, indoctrinate, and enable violence, persuasive techniques that were used to undermine youth's volitional capacity. Nonetheless, these techniques were also used by other members of the cell who came to reproduce the leader's model.

These psychological manipulation methods in turn could help the leader to attract, induce, and control group members. Of note, we identified that the use of these techniques was premediated (5, 24). Our results indicate that the psychological manipulation techniques were subtle and progressively played a role in convincing members of the cell that ideological violence was absolutely necessary to attain their goals.

These results are in line with various theories positing that social networks play an important role in the radicalization process leading to terrorism (24, 63). Particularly, these results provide some evidence for the psychosocial model of recruitment and violent mobilization (19, 20, 23). The evidence suggests that terrorist groups use psychological manipulation techniques to radicalize young people by creating psychological submission, political-religious indoctrination, and violent disinhibition. Moreover, the results of this study have theoretical implications, such as the adaptation of the manipulative dynamics by the recruiters to the social context and needs of young people. This involves focusing manipulation strategies on empathic and emotional aspects—such as making the individual feel identified, understood, and valued. At the same time, the more that coercive and abusive aspects are eliminated, the greater the chance of establishing an imperceptible and gradual persuasion without the involvement of violence or power.

This research has a number of potential practical implications. In particular, the results highlight the importance of paying attention to young people because they are a vulnerable target population for violent radicalization. Some common characteristics of the youth, such as the experience of extreme emotions, their sensitivity to threats, and their commitment with violence (3), potentially make them more vulnerable to these psychological manipulation strategies.

Despite adhering to rigorous research guidelines, this study has some limitations that may affect the interpretation of the results. First, this case study was only intended to describe the use of these techniques. Future studies should focus on the psychological effects of these techniques and how they facilitate the use of violence. Second, to facilitate the search for manipulation techniques, the interview designed by Cuevas (40) was adapted, marking the first time the instrument was used in this way. Future studies should be tested against other data sources and on other cases of group radicalization. Third, the sources used were secondary. Due to the death of most members of the cell, the only reliable information available is found in police reports. Thus, future studies should focus on other groups made up of young people, in which first-hand information can be obtained to confirm if techniques like those found in this research are used extensively.

Taken together, this study highlights the presence of psychological manipulation techniques (40) in the process of radicalization of the young members of the 17-A cell. Among these techniques, those aimed at controlling cognitions, environment, and emotions seem to be the most frequently used techniques and the most useful in generating group cohesion and disinhibiting ideological violence. Thus, we emphasized the need for comprehensive programs to prevent violent extremism among the youth. Therefore, it is crucial to provide young people with protective factors, such as self-control, empathy, education, employment, and basic attachment to society (64). In brief, there is a great need for governments and organizations that work with young people to be active where radicalization agents may be present. Any space of influence in which the system, the institutions, or adults are not proactively present can be a fertile ground for terrorist recruitment (65).

## Data Availability Statement

The original contributions presented in this study are available and can be requested from the corresponding authors.

## Author Contributions

IG and MM: conceptualization, investigation, methodology, formal analysis, writing—original draft, and writing—review and editing. RL and HT: methodology, formal analysis, and writing—review and editing. All authors contributed to the article and approved the submitted version.

## Funding

This work was funded by Centro Mixto UGR-MADOC (18/16 CEMIX UGR-MADOC), MCIN/AEI/10.13039/501100011033/ and European Union “NextGenerationEU”/PRTR under Grant PID2020-116646RB-I00.

## Conflict of Interest

The authors declare that the research was conducted in the absence of any commercial or financial relationships that could be construed as a potential conflict of interest.

## Publisher's Note

All claims expressed in this article are solely those of the authors and do not necessarily represent those of their affiliated organizations, or those of the publisher, the editors and the reviewers. Any product that may be evaluated in this article, or claim that may be made by its manufacturer, is not guaranteed or endorsed by the publisher.
